# Supplementary material for: Faster Time to Treatment Decision of Viscoelastic Coagulation Test Results through Improved Perception with the Animated Visual Clot: A Multicenter Comparative Eye-Tracking Study
Source: Diagnostics (Basel). 2022 May 19;12(5):1269. doi: 10.3390/diagnostics12051269 (PMC9140857; doi:10.3390/diagnostics12051269)

A representation of the study setup including the evaluated recording of a Visual Clot with normal findings, displayed in the software Pupil Player version 5.5. The pink rectangle represents the drawn area of interest. The green dot with the pink line in the center represents the eye movement recorded by the eye tracker.

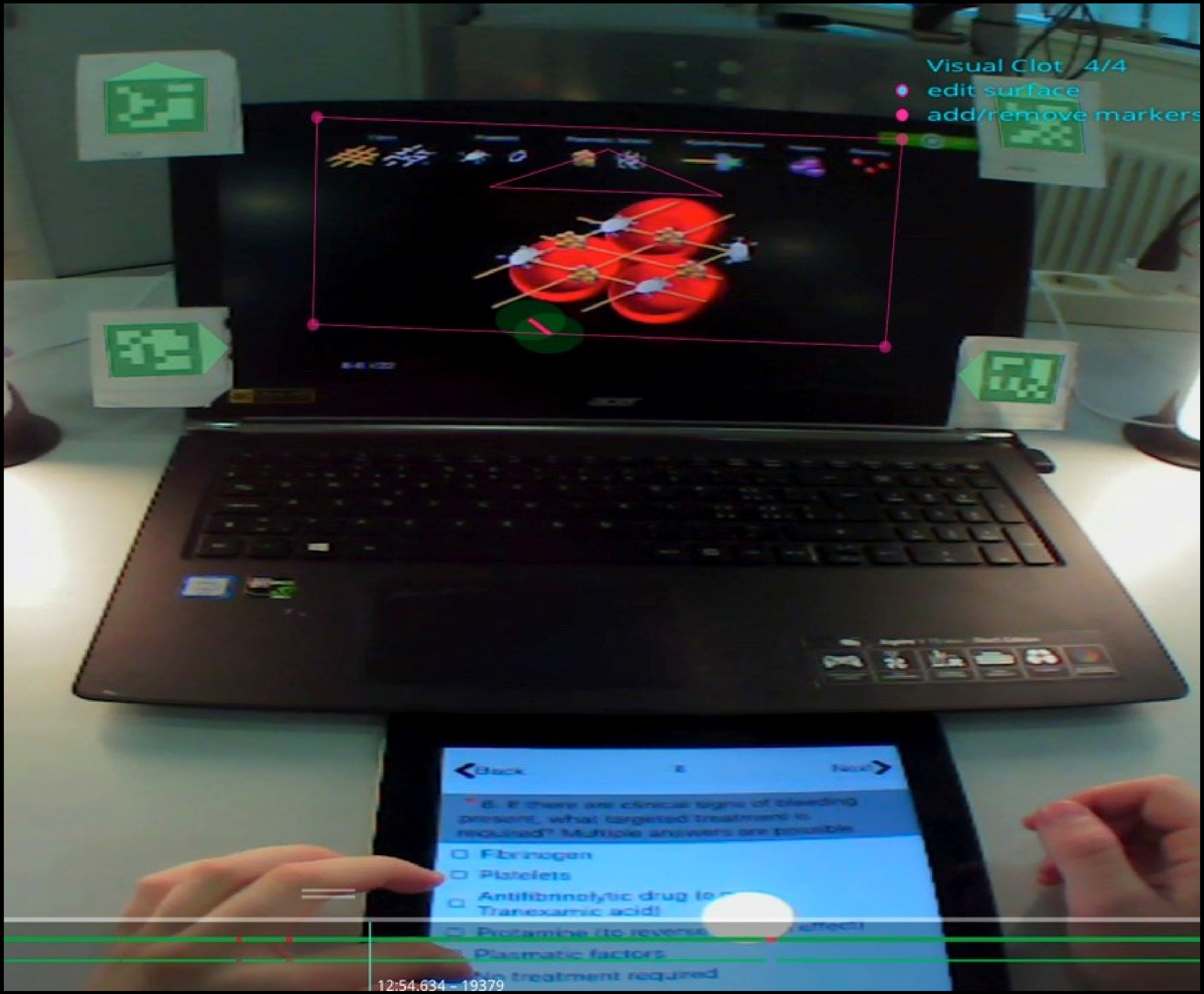

A representation of the study setup including the evaluated recording of a ROTEM temogram with normal findings, displayed in the software Pupil Player version 5.5. The pink rectangle represents the drawn area of interest. The green dot with the pink line in the center represents the eye movement recorded by the eye tracker.

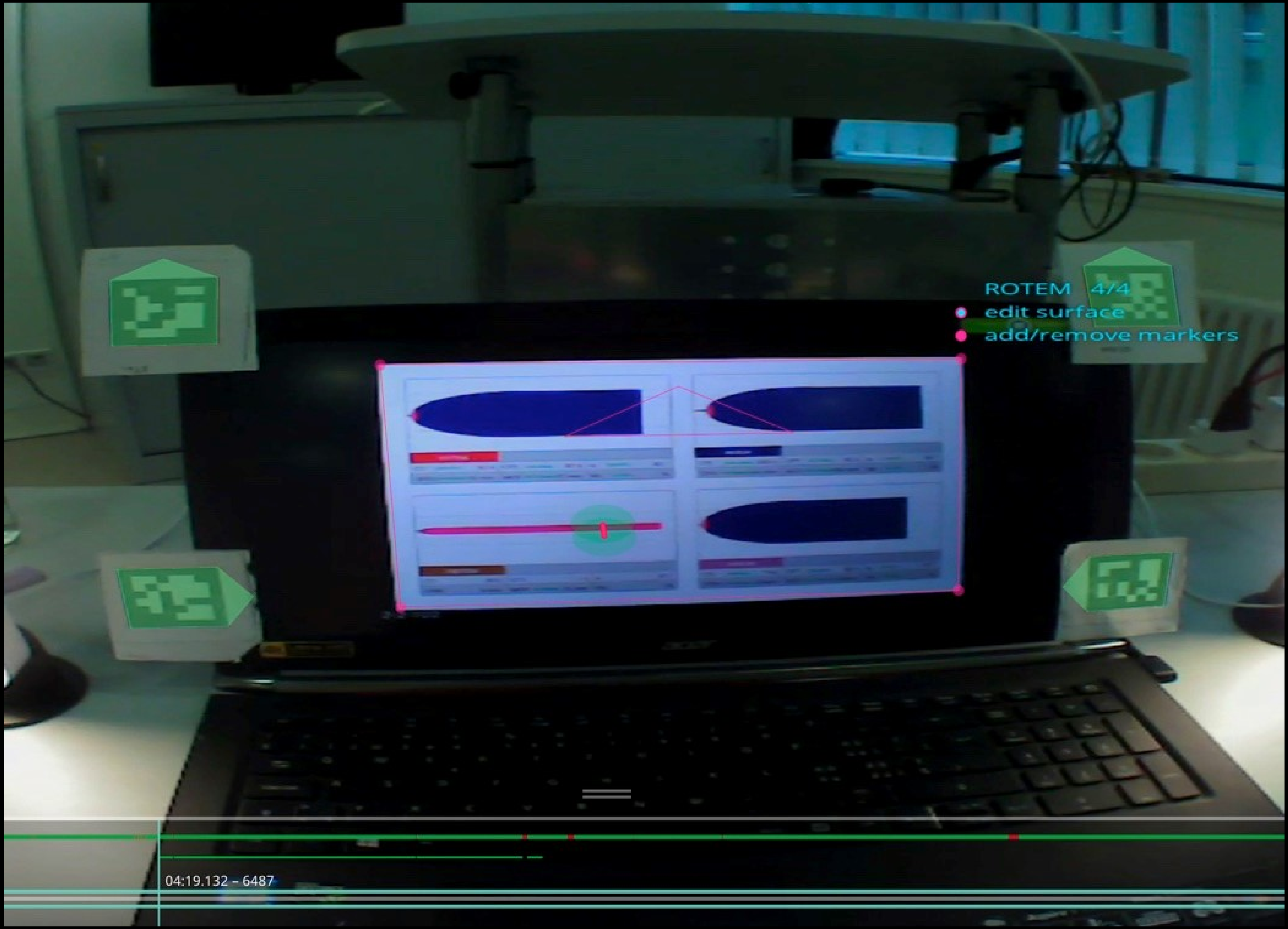

Supplement: Supplementary file 1 [file diagnostics-12-01269-s001.zip › Figure S1_VisualClot_ROTEM_AOI.pdf]
