# Supplementary material for: Faster Time to Treatment Decision of Viscoelastic Coagulation Test Results through Improved Perception with the Animated Visual Clot: A Multicenter Comparative Eye-Tracking Study
Source: Diagnostics (Basel). 2022 May 19;12(5):1269. doi: 10.3390/diagnostics12051269 (PMC9140857; doi:10.3390/diagnostics12051269)

# Case 1: Normal

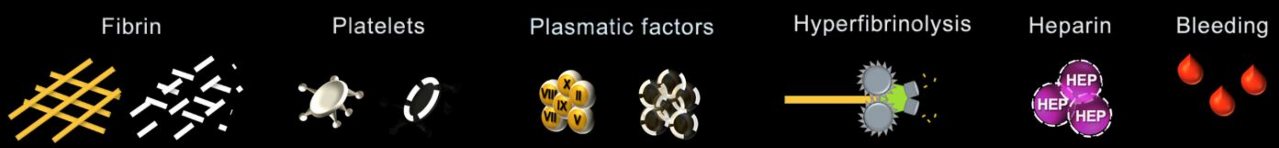


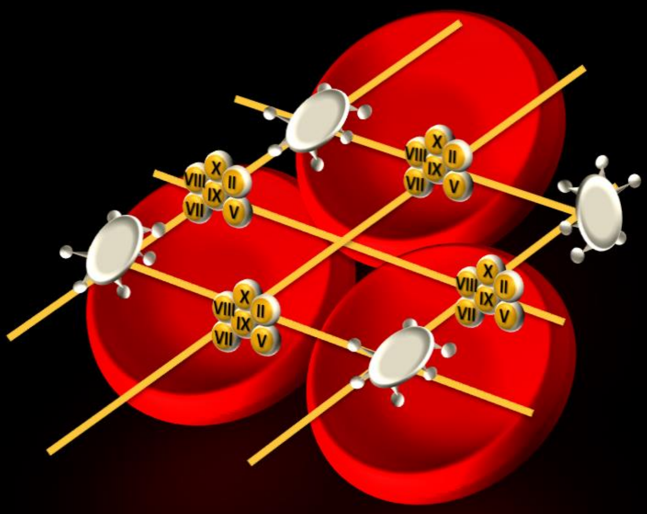

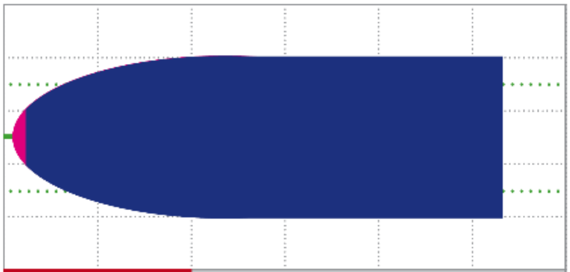

| EXTEM | | | | | |
|---|---|---|---|---|---|
| CT: | 67 s | CFT: | 87 s | α: | 63 ° |
| CFR: | 54 mm | MCF: | 57 mm | ML: | - % |

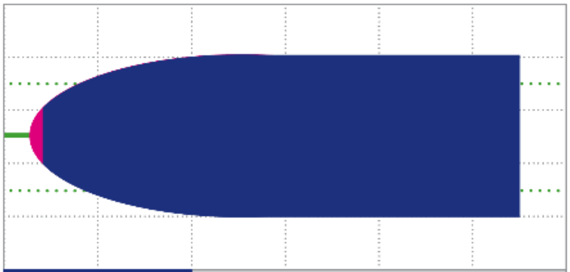

| INTEM | | | | | |
|---|---|---|---|---|---|
| CT: | 200 s | CFT: | 85 s | α: | 64 ° |
| CFR: | 54 mm | MCF: | 58 mm | ML: | - % |

1

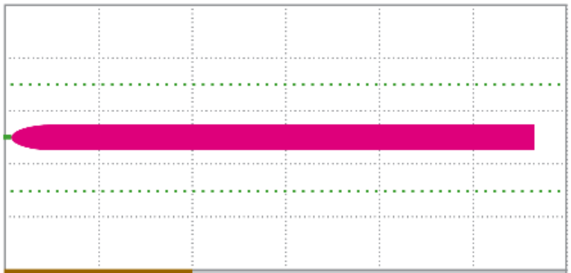

| FIBTEM | | | | | |
|---|---|---|---|---|---|
| CT: | 66 s | CFT: | - s | α: | 57 ° |
| CFR: | 9 mm | MCF: | 15 mm | ML: | - % |

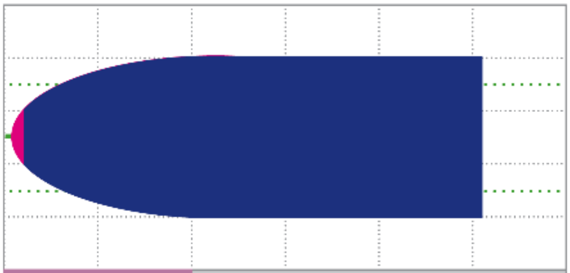

| APTEM | | | | | |
|---|---|---|---|---|---|
| CT: | 74s | CFT: | 89 s | α: | 62° |
| CFR: | 53mm | MCF: | 61mm | ML: | - % |

# Case 2: Fibrin deficiency

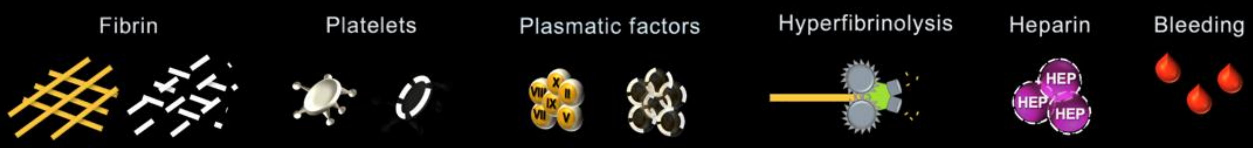


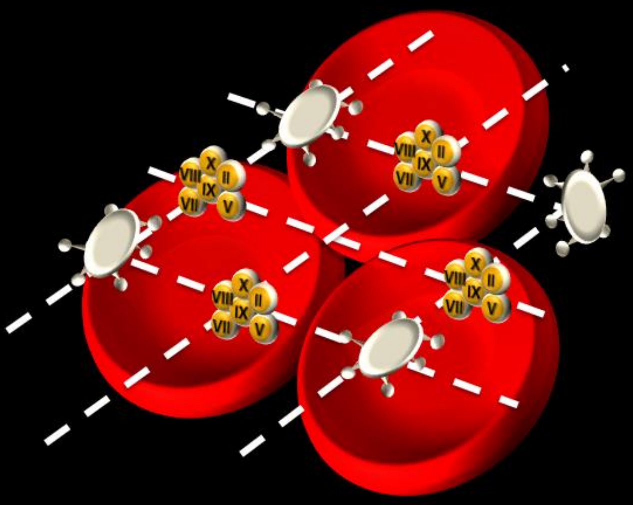


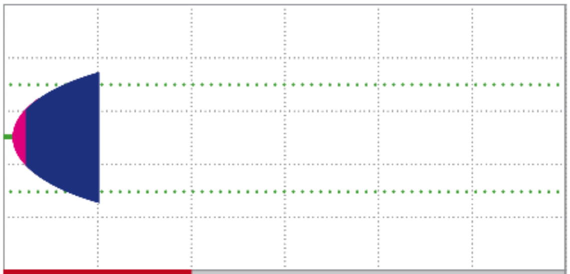

| EXTEM | | | | | |
|---|---|---|---|---|---|
| CT: | 67 s | CFT: | 87 s | α: | 63 ° |
| A10: | 45 mm | MCF: | 46 mm | ML: | - % |

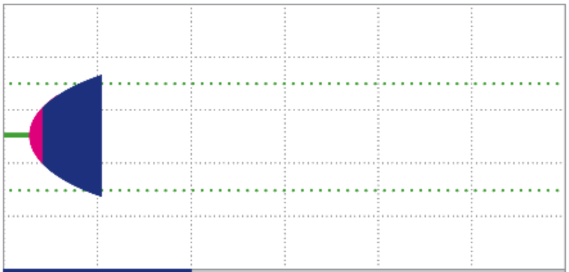

| INTEM | | | | | |
|---|---|---|---|---|---|
| CT: | 200 s | CFT: | 85 s | α: | 64 ° |
| A10: | 44 mm | MCF: | 45 mm | ML: | - % |

1

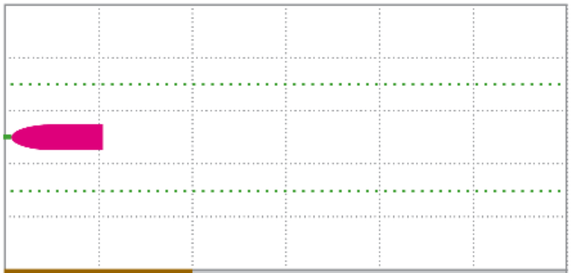

| FIBTEM | | | | | |
|---|---|---|---|---|---|
| CT: | 66 s | CFT: | - s | α: | 57 ° |
| A10: | 14 mm | MCF: | 15 mm | ML: | - % |

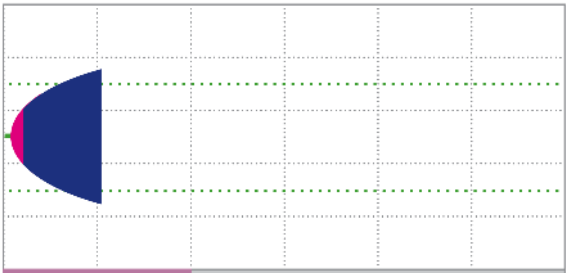

| APTEM | | | | | |
|---|---|---|---|---|---|
| CT: | 74s | CFT: | 89 s | α: | 62° |
| A10: | 47 mm | MCF: | 48 mm | ML: | - % |

# Case 3: Platelets deficiency

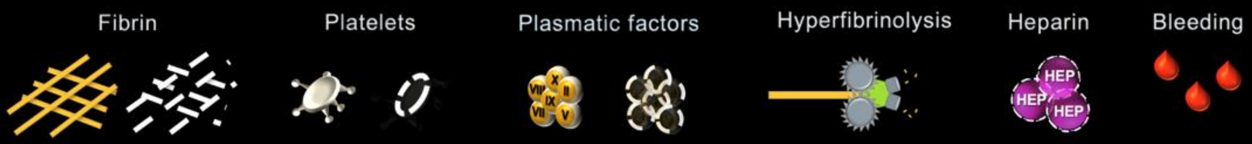


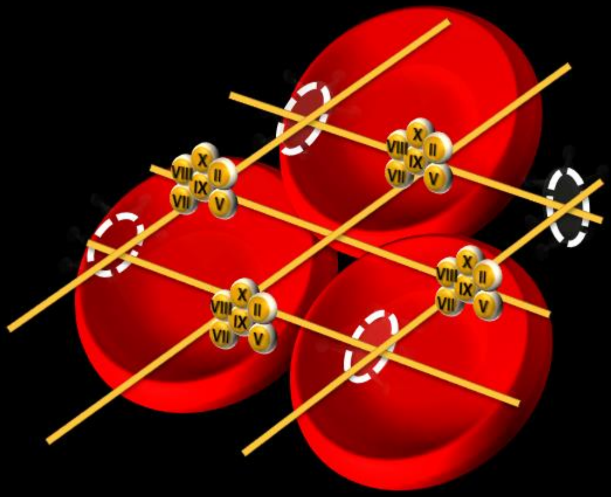

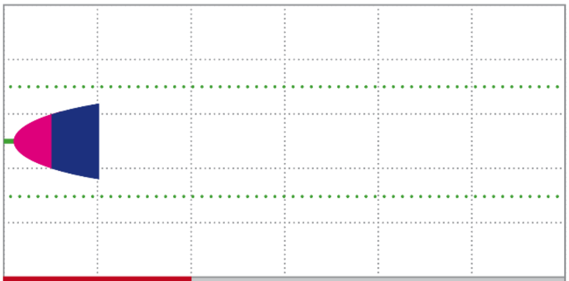

| EXTEM | | | | | |
|---|---|---|---|---|---|
| CT: | 81 s | CFT: | 354 s | α: | 76 ° |
| A10: | 25 mm | MCF: | 26 mm | ML: | - % |

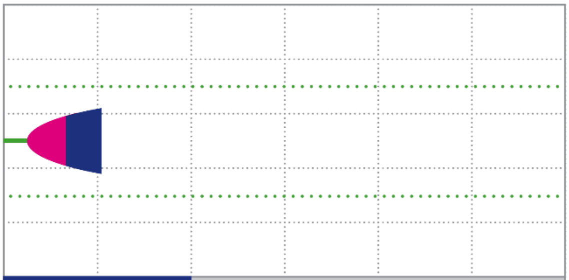

| INTEM | | | | | |
|---|---|---|---|---|---|
| CT: | 206 s | CFT: | 382 s | α: | 72 ° |
| A10: | 22 mm | MCF: | 23 mm | ML: | - % |



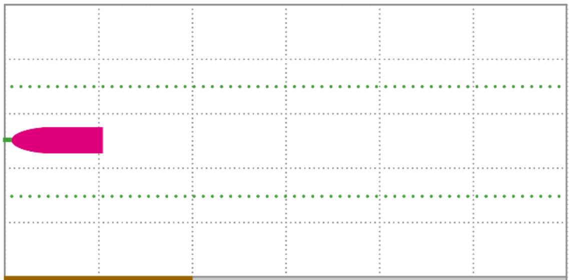

| FIBTEM | | | | | |
|---|---|---|---|---|---|
| CT: | 80 s | CFT: | - s | α: | - ° |
| A10: | 14 mm | MCF: | 15 mm | ML: | - % |

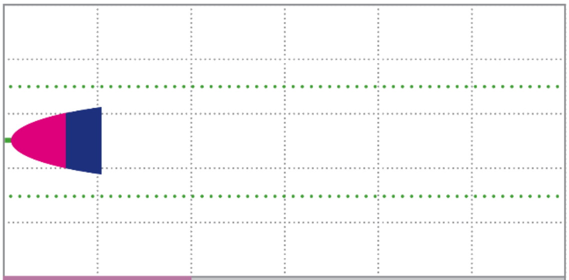

| APTEM | | | | | |
|---|---|---|---|---|---|
| CT: | 94 s | CFT: | 347 s | α: | 77 ° |
| A10: | 26 mm | MCF: | 27 mm | ML: | - % |

# Case 4: Plasmatic factors deficiency

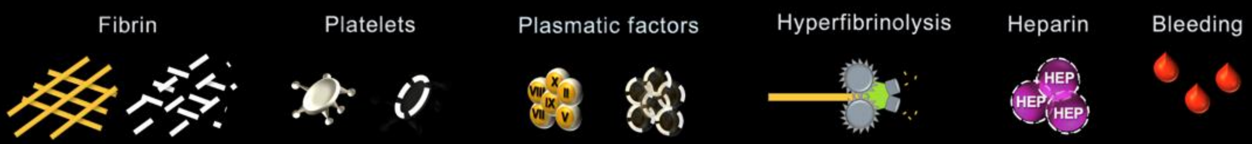


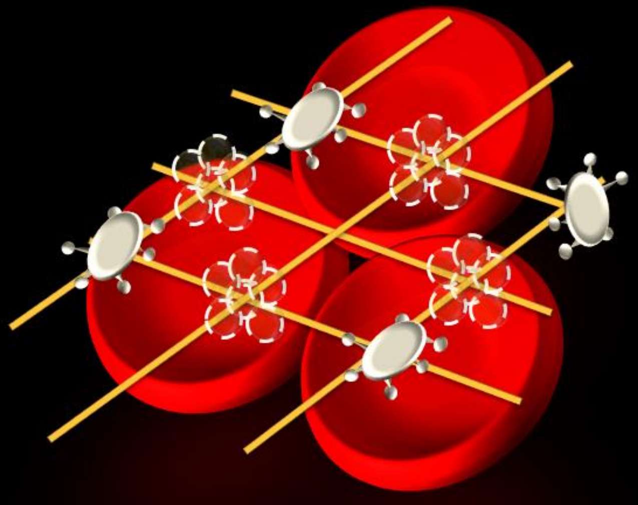

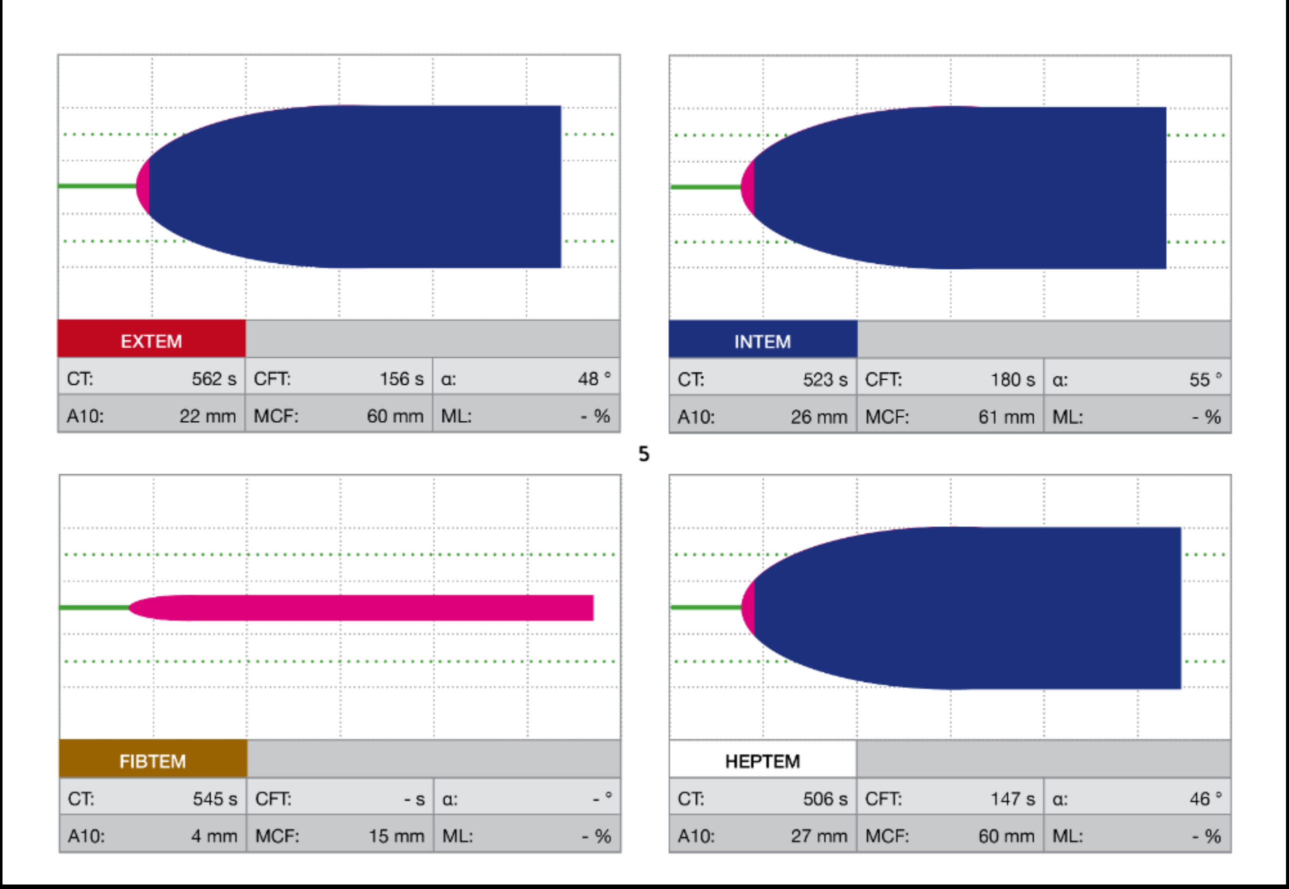

# Case 5: Hyperfibrinolysis

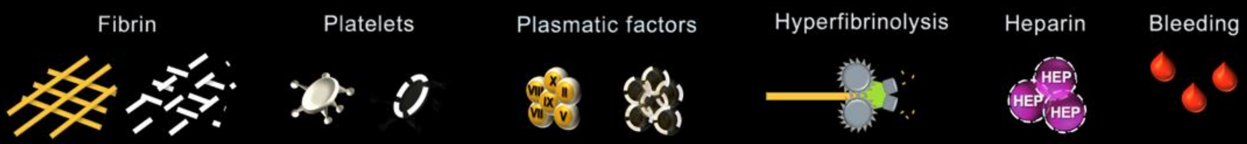


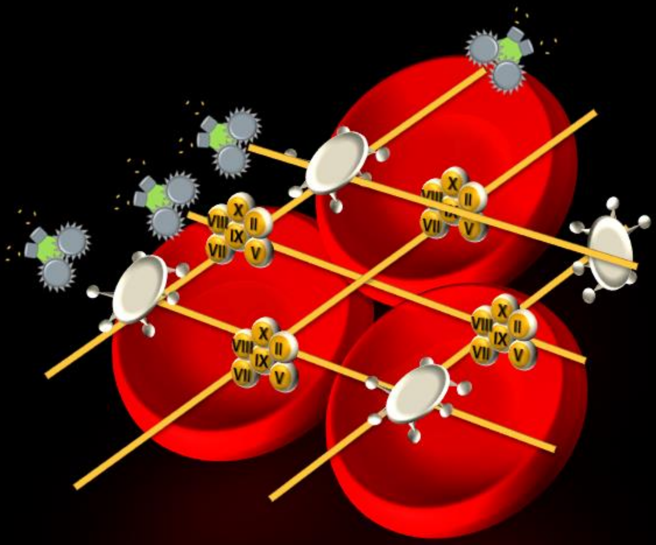

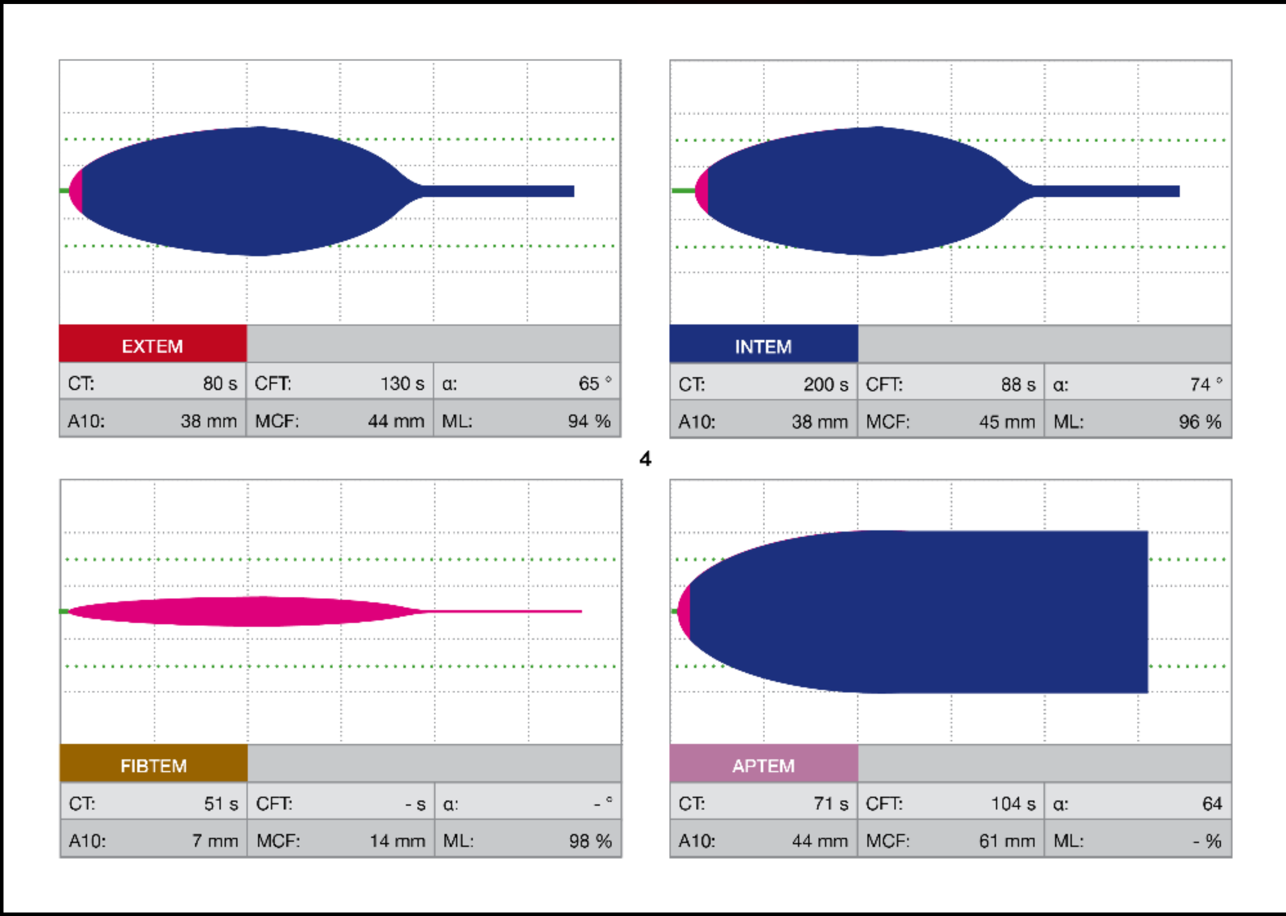

# Case 6: Heparin effect

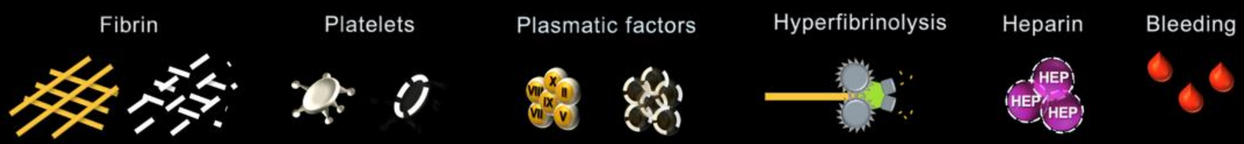


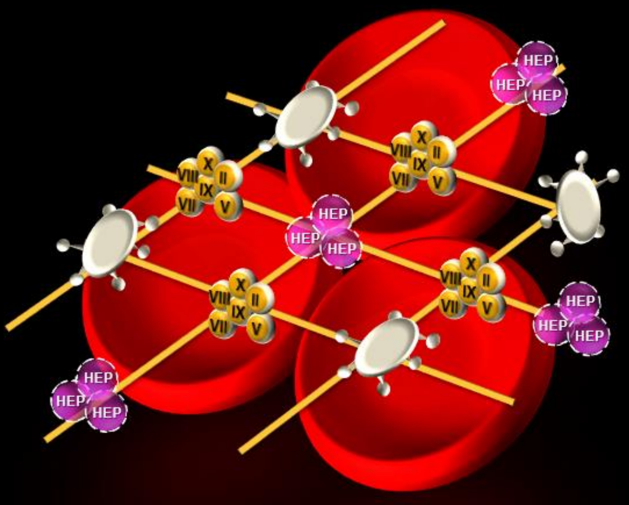


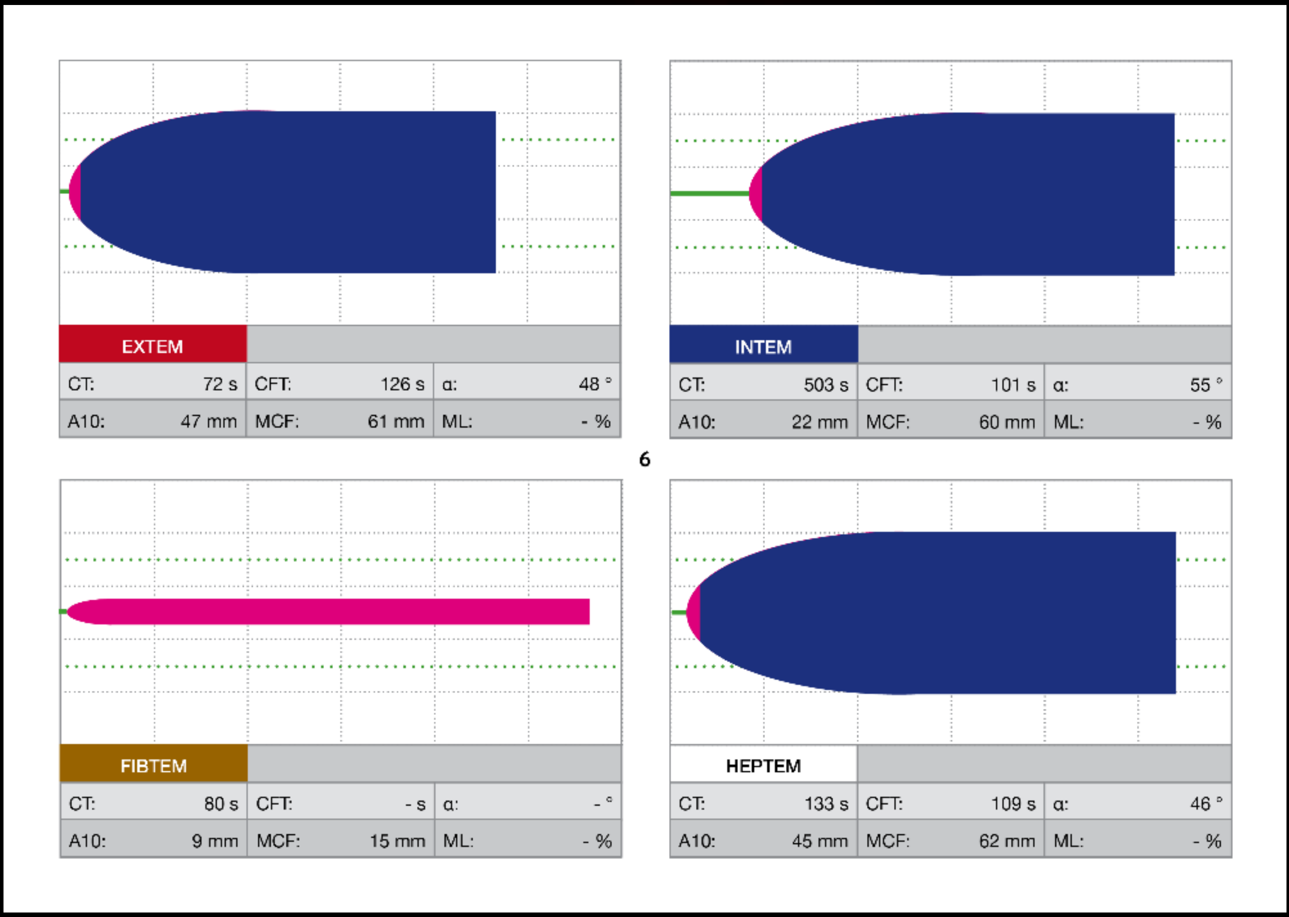

# Case 7: Hypercoagulability

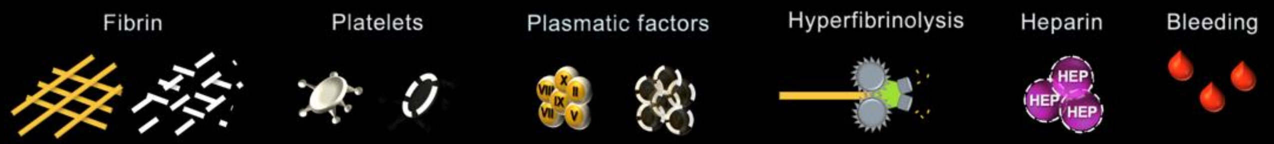


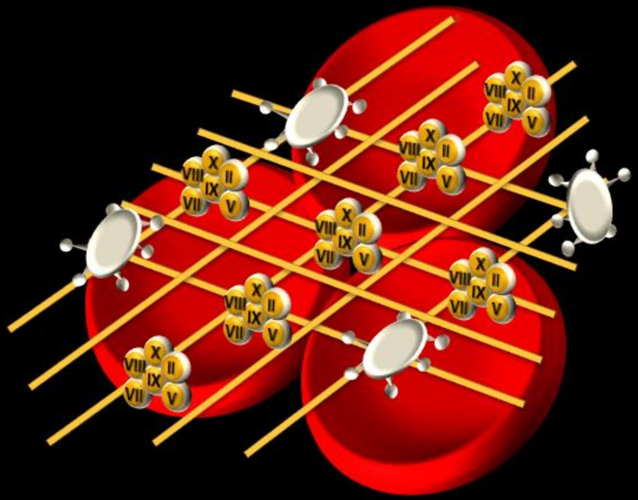

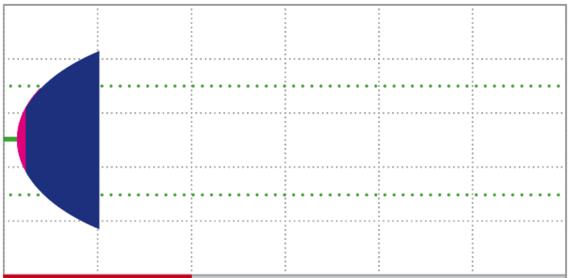

| EXTEM | | | | | |
|---|---|---|---|---|---|
| CT: | 99 s | CFT: | 56 s | α: | 48 ° |
| A10: | 64 mm | MCF: | 66 mm | ML: | - % |

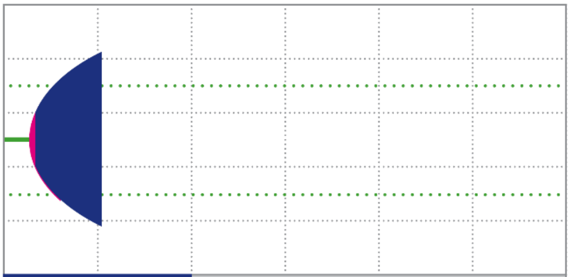

| INTEM | | | | | |
|---|---|---|---|---|---|
| CT: | 212 s | CFT: | 50 s | α: | 55 ° |
| A10: | 63 mm | MCF: | 64 mm | ML: | - % |

**7**

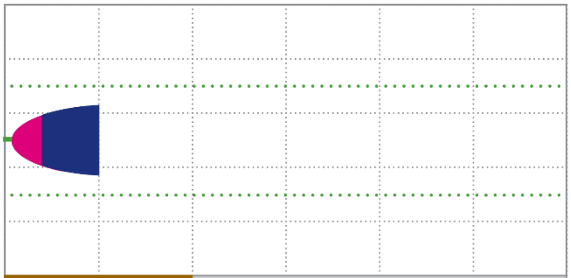

| FIBTEM | | | | | |
|---|---|---|---|---|---|
| CT: | 45 s | CFT: | 269 s | α: | - ° |
| A10: | 28 mm | MCF: | 29 mm | ML: | - % |

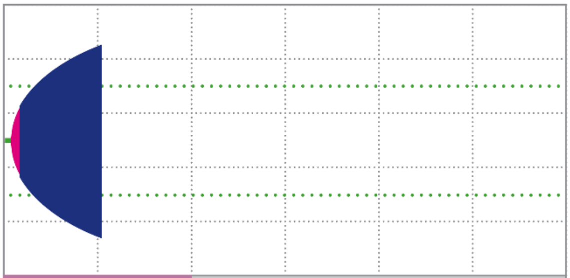

| APTEM | | | | | |
|---|---|---|---|---|---|
| CT: | 94 s | CFT: | 67 s | α: | 46 ° |
| A10: | 63 mm | MCF: | 65 mm | ML: | - % |

# Case 8: Combined plasmatic factors, fibrin and platelets deficiency

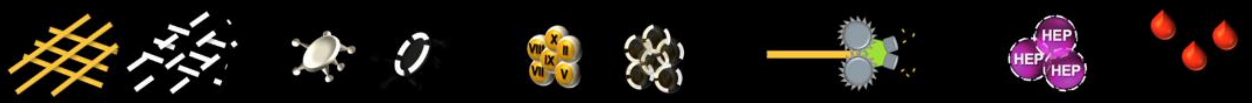


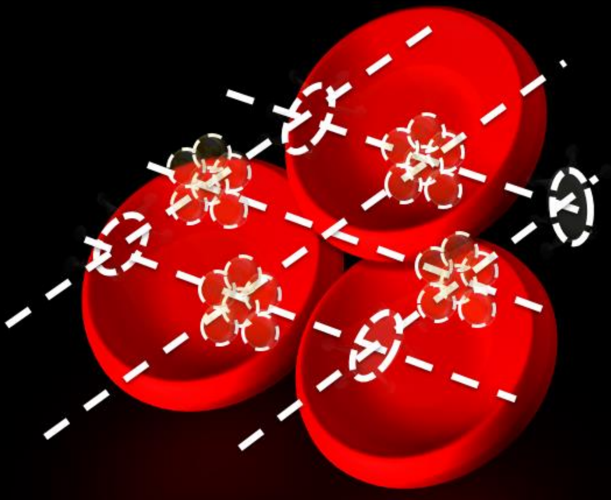

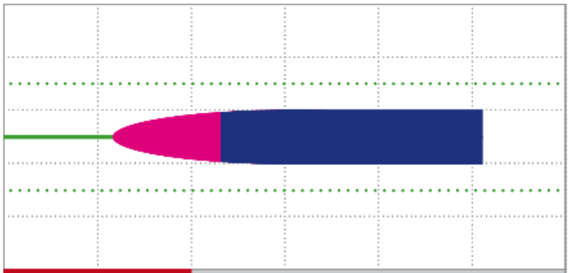

| EXTEM | | | | | |
|---|---|---|---|---|---|
| CT: | 735 s | CFT: | 723 s | α: | 48 ° |
| A10: | - mm | MCF: | 21 mm | ML: | - % |

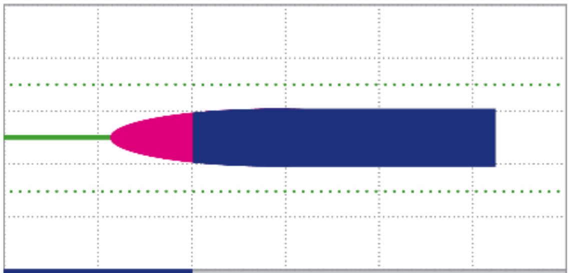

| INTEM | | | | | |
|---|---|---|---|---|---|
| CT: | 711 s | CFT: | 532 s | α: | 55 ° |
| A10: | - mm | MCF: | 22 mm | ML: | - % |



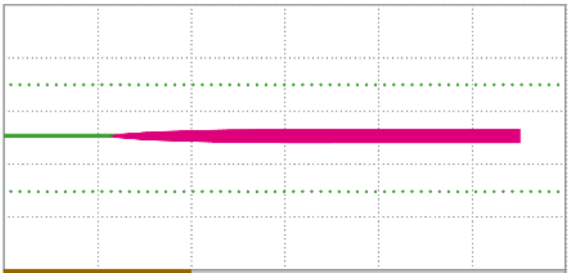

| FIBTEM | | | | | |
|---|---|---|---|---|---|
| CT: | 898 s | CFT: | - s | α: | - ° |
| A10: | - mm | MCF: | 7 mm | ML: | - % |

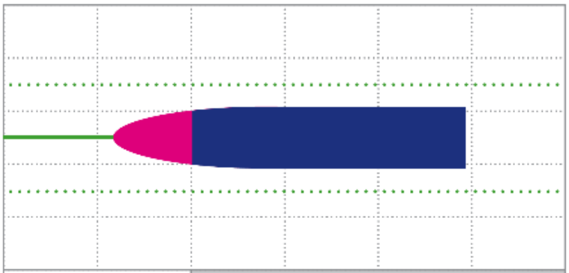

| HEPTEM | | | | | |
|---|---|---|---|---|---|
| CT: | 717 s | CFT: | 504 s | α: | 46 ° |
| A10: | - mm | MCF: | 23 mm | ML: | - % |

# Case 9: Combined plasmatic factors and fibrin deficiency, and hyperfibrinolysis

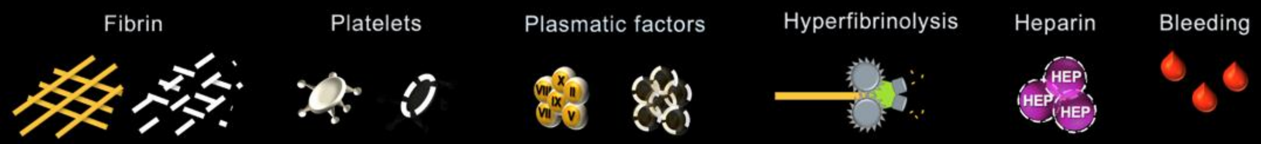


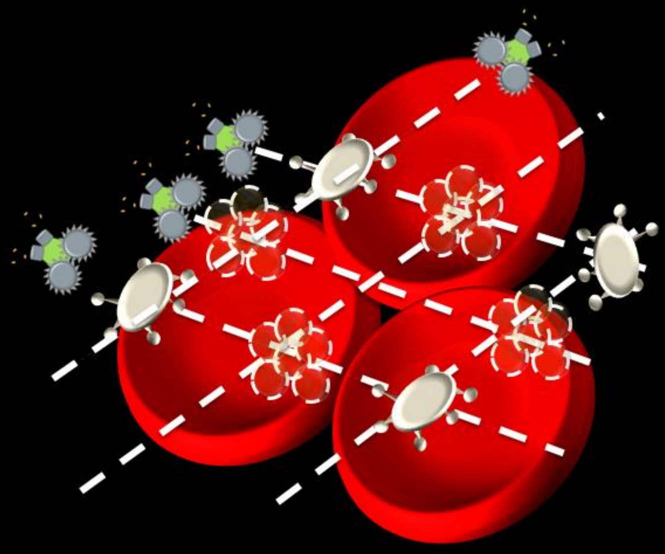

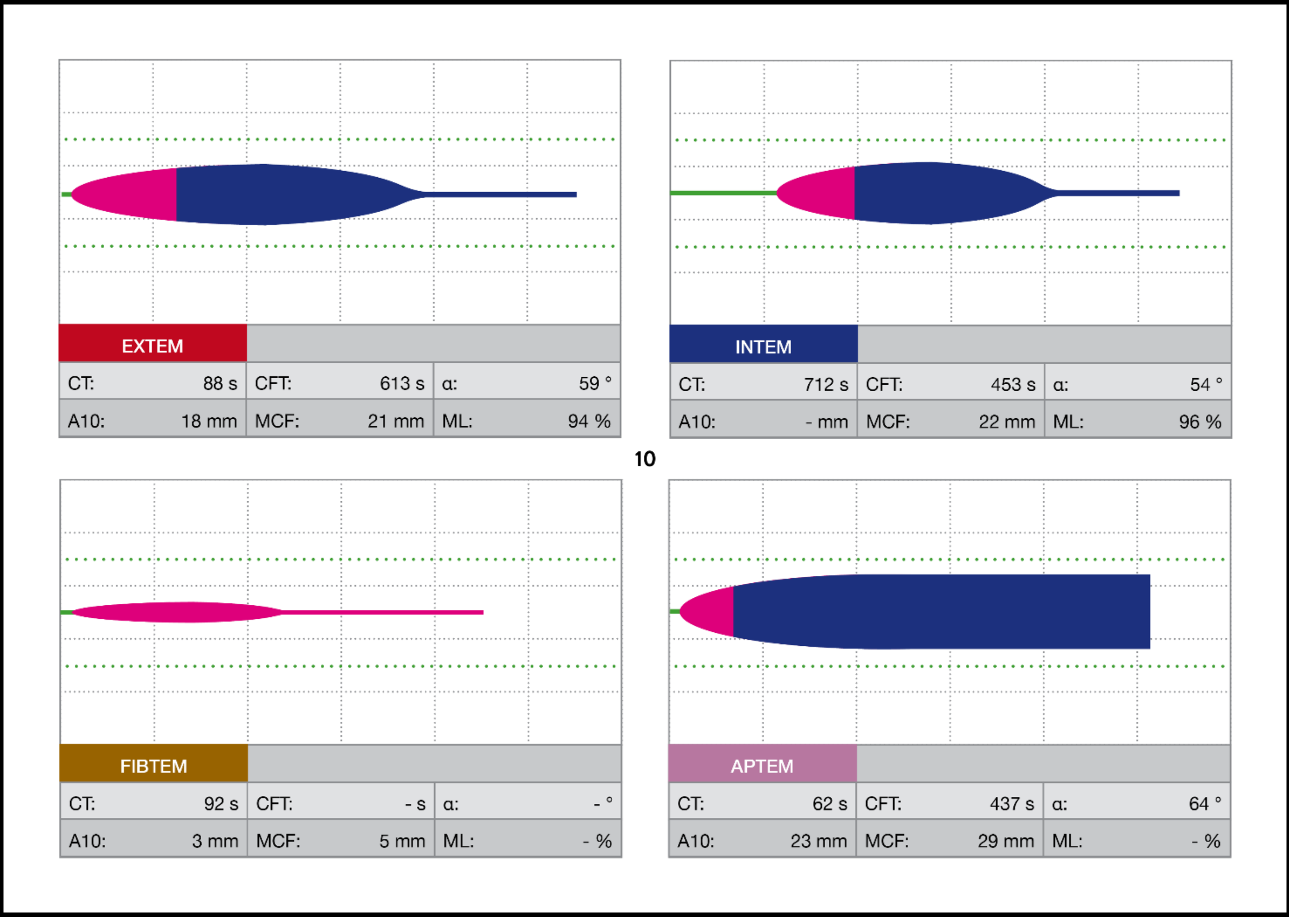

Supplement: Supplementary file 1 [file diagnostics-12-01269-s001.zip › Figure S2_Cases_1-9_ROTEM-VC_20220321.pdf]
