# Supplementary material for: Faster Time to Treatment Decision of Viscoelastic Coagulation Test Results through Improved Perception with the Animated Visual Clot: A Multicenter Comparative Eye-Tracking Study
Source: Diagnostics (Basel). 2022 May 19;12(5):1269. doi: 10.3390/diagnostics12051269 (PMC9140857; doi:10.3390/diagnostics12051269)
Supplement: Supplementary file 1 [file diagnostics-12-01269-s001.zip › Table S1_VisualClotAlgorithm.pdf]

Faster Time to Treatment Decision of Viscoelastic Coagulation Test Results through Improved Perception with the Animated Visual Clot: A Multicenter Comparative Eye-Tracking Study

Clara Castellucci[1], Julia Braun[2]; Sadiq Said[1]; Tadzio Raoul Roche[1]; Christoph B Nöthiger[1]; Donat R Spahn[1]; David W Tscholl[1;] Samira Akbas[1]

Table S1: Visual Clot algorithm*. MCF=Maximum Clot Firmness, ML=Maximum Lysis.

Fibrin deficiency = true	IF "MCF FIBTEM" < 9 mm
Thrombocyte deficiency = true	IF "MCF FIBTEM" 9-25 mm AND "MCF EXTEM" < 40mm OR "MCF INTEM" < 40mm<br>OR<br>IF "MCF FIBTEM" > 25mm AND "MCF EXTEM" < 50mm OR "MCF INTEM" < 50mm<br>OR<br>IF "MCF FIBTEM" < 9mm AND "MCF EXTEM" < 30mm OR "MCF INTEM" < 30mm<br>OR<br>IF "ML INTEM" > 15% OR "ML EXTEM" > 15% OR "ML FIBTEM" > 15% AND "MCF APTEM" < 30mm
Plasmatic factors deficiency = true	IF "CT EXTEM" > 79sec OR "CT INTEM" > 240sec AND "Fibrin deficiency" = false AND "Thrombocyte deficiency" = false AND "Hyperfibrinolysis" = false AND "Hypercoagulability" = false AND "Heparin effect" = false<br>OR<br>IF "CT EXTEM" >100sec OR "CT INTEM" > 260sec.
Hyperfibrinolysis = true	IF "ML EXTEM" > 15% OR "ML INTEM" > 15% OR "ML FIBTEM" > 15% AND "ML APTEM" < 15%
Hypercoagulability = true	IF "MCF EXTEM" > 72mm or "MCF INTEM" > 72mm
Heparin effect = true	IF "CT INTEM" > 240sec AND "CT HEPTEM" < 240sec

*This algorithm was published before in:
Wetli DJ, Bergauer L, Nöthiger CB, Roche TR, Spahn DR, Tscholl DW, Said S. Improving Visual-Patient-Avatar Design Prior to Its Clinical Release: A Mixed Qualitative and Quantitative Study. Diagnostics (Basel). 2022 Feb 21;12(2):555. doi: 10.3390/diagnostics12020555. PMID: 35204644; PMCID: PMC8871093.
It is provided as a courtesy to the reader for completeness of information.
